# Study of the Peripheral Nerve Fibers Myelin Structure Changes during Activation of Schwann Cell Acetylcholine Receptors

**DOI:** 10.1371/journal.pone.0158083

**Published:** 2016-07-25

**Authors:** Ekaterina E. Verdiyan, Elvin S. Allakhverdiev, Georgy V. Maksimov

**Affiliations:** 1 Faculty of Biology, M.V. Lomonosov Moscow State University, Moscow, Russia; 2 Faculty of Fundamental Medicine, M.V. Lomonosov Moscow State University, Moscow, Russia; University of Hyderabad, INDIA

## Abstract

In the present paper we consider a new type of mechanism by which neurotransmitter acetylcholine (ACh) regulates the properties of peripheral nerve fibers myelin. Our data show the importance of the relationship between the changes in the number of Schwann cell (SC) acetylcholine receptors (AChRs) and the axon excitation (different intervals between action potentials (APs)). Using Raman spectroscopy, an effect of activation of SC AChRs on the myelin membrane fluidity was investigated. It was found, that ACh stimulates an increase in lipid ordering degree of the myelin lipids, thus providing evidence for specific role of the “axon-SC” interactions at the axon excitation. It was proposed, that during the axon excitation, the SC membrane K^+^- depolarization and the Ca2+—influx led to phospholipase activation or exocytosis of intracellular membrane vesicles and myelin structure reorganization.

## Introduction

It is known, that myelin sheath of peripheral nerve fibers consists of multiple layers of Schwann cell (SC) membranes, wrapped around the axon [1, 2]. Emerging data suggests that specific interactions between axon and SC govern neuronal function [1]. SC responds to axonal signals of neuron in several ways, including signaling mediated by different neurotransmitters [3]. Villegas with colleagues was the first who investigated this type of signaling on nerve fibers of invertebrates [4]. They demonstrated the presence of acetylcholine receptors (AChRs) on SC membrane, exposed especially to intercellular space between axon and the surrounding SC [5]. More recently, it has been shown that SCs of vertebrates are also associated with AChRs [6–8]. In addition, the number of AChRs was shown to change during the nerve fibers function [7]. It was proposed that the changes of ^3^H-bungarotoxin binding to nerve during the nerve excitation initiate the transformation of "inactive" forms of acetylcholine receptor of SC membrane to "active" ones, which depend on the structural alterations in paranodal space axolemma proteins [8]. This study continued an investigation of the changes of SC AChRs number in regulation of the myelin membrane fluidity.

As the structure of myelinated nerve fiber is quite complex, there is a problem related to the absence of experimental methods to study the changes in intact cell without its labeling [2]. Here we applied a non-invasive method of Raman spectroscopy (RS) to study the “axon-SC” signaling mediated by activation of SC AChRs for investigation of the myelin lipid degree of order [9,10]. Long time has passed since the first Raman spectra from the myelin sheath of sciatic nerve were recorded [11–15]. Further investigation revealed that myelin of nerve fibers contains carotenoid molecules on its membrane which have predominantly radial-like orientation [9,10,12]. Probably, if the carotenoid molecule changes their conformation due to the changes in membrane potential, further it may create tensions in the conformation surrounding lipids of the nerve fiber. Thus, carotenoids can be mediators of voltage adjustment of myelin and are able to regulate interactions of ligands with lipid bilayer [12]. After that, different variations of RS were developed and applied to assess the molecular structure of myelin upon different pathologies [13].

The objective of this work was to detect the relationship between the appearance of the active AChRs on the plasma membrane of Schwann cell and changes of the state of the fatty acids "tails" of phospholipids and carotenoid molecules in the myelin lipid bilayer. A new mechanism of regulation of the nerve excitation propagation, when the state of myelin in viscosity is determined by the activation of SC AChR, is suggested.

## Materials and Methods

### Nerve fibers preparation

All experiments were performed on myelinated nerve fibers of frog *Rana temporaria*. All experiments on animals (we used 60 animals aged twelve months from the vivarium of the Faculty of Biology, M.V. Lomonosov Moscow State University) were carried out in accordance with the animal care regulations of the M.V. Lomonosov Moscow State University. The protocol was approved by the Bioethics Committee of the Faculty of Biology, M.V. Lomonosov Moscow State University. Prior to experiments male frogs were maintained in containers with some water at +5 C without feeding. Animals were anesthetized using solution of propofol (50 mg/kg) administered intraceolomically, which ensured the deep anesthesia condition prior to euthanasia. Anesthetized animals were decapitated with the following pithing of the brain.

Nerves were dissected and then incubated in Ringer buffer solution consisting of 100 mM NaCl, 2 mM KCl, 1.08 mM CaCl_2_, and 10 mM HEPES (pH 7.4) for at least 30 min. Single nerve fibers for RS measurements were carried out by splitting each nerve. Experiments were done at room temperature (20–22°C). Nerve fibers were placed on a glass bottom of a FluoroDish (World Precision Instruments, USA) filled with Ringer buffer solution (or solution containing ACh), and then covered by a glass coverslip. Acetylcholine bromide (Sigma) solution was prepared by dissolving the powder in Ringer buffer [7,8].

### Isotopic analysis

The isotopic analysis was used for investigation of the nerve fiber binding ^3^H—bungarotoxin (10 μCu/ml), ^3^H- acetylcholine (5 μCu/ml) and ^3^H- choline chloride (5 μCu/ml) (Amersham Biosciences, USA). The nerves were incubated in Ringer solution containing the label. Then, the nerves were washed, dried, weighed and the nerve radioactivity was tested using liquid scintillation method (Mark-2 or Intertechnique, (France)). The nerves were homogenized and placed in the plastic tube with 5 ml of liquid scintillator JS-106. Label content was calculated by the formula *C = A/M∙a*, where *A* is the count rate (cpm), *M*—nerve weight (in mg), and *a* is the known amount of the corresponding count rate isotope (cpm /mol) [7,8].

### Raman spectroscopy

For Raman spectroscopy study of nerve fibers, we used the confocal Raman spectrometer NTEGRA Spectra (NT-MDT, Russia) with a 532-nm laser excitation. Raman spectra were recorded by focusing the laser beam on the nerve fiber myelin surface through the ×40 objective with NA 0.6 (LUCPlanFL N, Olympus, Japan). Laser power was set to 0.8 mW and integration time for each spectrum was 50–60 s. Spectrometer has a grating with 600 lines/mm and entrance slit set to 100 μm. Raman scattered light was collected in a backscattering mode and detected with a CCD camera (1024 × 256 pixels, -50°C). Subsequent Raman spectra baseline subtraction and calculation of Raman peak intensities were made using OriginPro 2015 software (OriginLab Corporation, USA) [9,16].

## Results and Discussion

### The Schwann cell ^3^H- bungarotoxin and ^3^H- acetylcholine binding during axon stimulation with different number and interval between the conducting action potentials series along the axon

**Fig 1:**
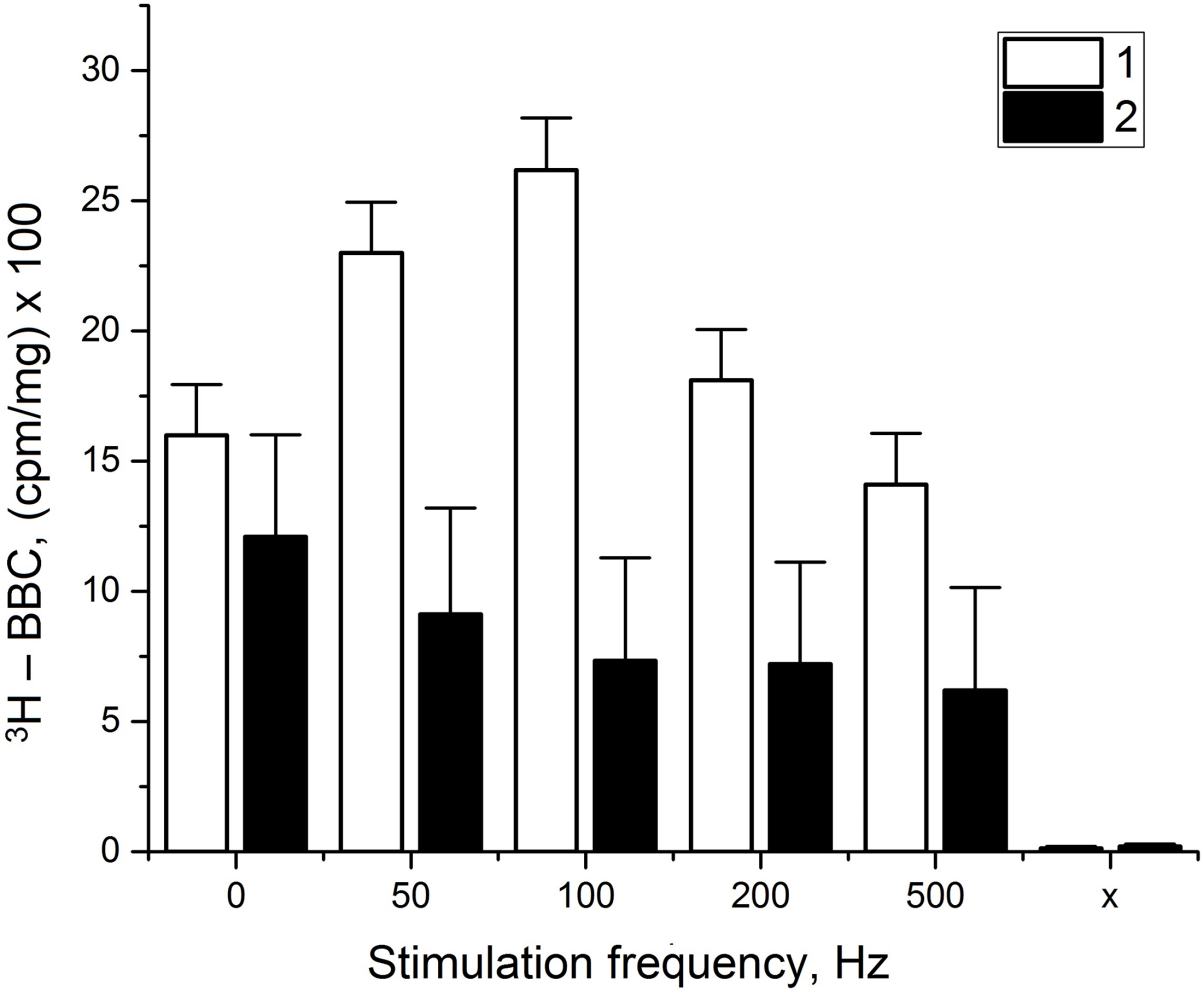
Dependence of specific 3H- bungarotoxin binding by Schawann cell myelin nerve fiber on the stimulation frequency. Experiments demonstrate specific ^3^H-bungarotoxin binding by Schawann cell myelin nerve fiber during the different rhythmic excitation frequency (white bars, 1) and during incubation with nicotine (10^−6^ M) (black bars, 2). “0”–control; the time of nerve fiber incubation was 60 min, and time of nerve stimulation was 10 min. Here, BBC—bungarotoxin binding content; significant difference between points (p < 0.05).

The changes of ^3^H- bungarotoxin and ^3^H- acetylcholine binding during nerve excitation (changes of the number of AChRs in a SC membrane) during different number of action potentials and interval between the conducting action potentials (APs) series along the axon were studied. In earlier experiments, we have found that non myelin nerve fibers ^3^H- bungarotoxin binding could be specific—from five to ten minutes and for myelin nerve fibers—up to hour [6–8]. In this experiments were found that the specific binding ^3^H- bungarotoxin (^3^H-BT) depends on the different number of APs and the interval between single APs (Fig 1): when the interval between axon AP decreases, the SC ^3^H- bungarotoxin binding increases to maximum (at stimulation frequency—50–100 Hz a difference between black and white bars is significant (p <0.05)), but it decreases upon small intervals between APs. So, at the optimal nerve functional regime, the decreasing interval between axon APs propagated and the SC AChRs numbers increases.

It was shown that the external application of nicotine to the resting nerve fiber is able to reproduce the long-lasting effects on the SC membrane potential, but had no effect on the resting and action potentials of the axon [4]. To test the specificity of this process, we kept the nerve fibers in a solution containing the ^3^H- bungarotoxin and nicotine, which is a toxin concurrent to AChR. These data show the presence of AChRs of the nicotinic type in the SC, and give support to the hypothesis on the role of the long-lasting Schwann cell hyperpolarization’s in myelin structure changes during the axon conduction of APs.

This data (Fig 1) reveals the important role of the relations between the change in number of SC AChRs and the axon excitation (different interval between single APs). Possibly, activation of SC during axon excitation depends on the axon K^+^—flux and the extracellular Ca^2+^ redistribution [17]. All this processes leads to stimulation of Ca^2+^- and K^+^ -influx in SC and output of the ACh in nerve fiber paranodal space [18].

**Fig 2:**
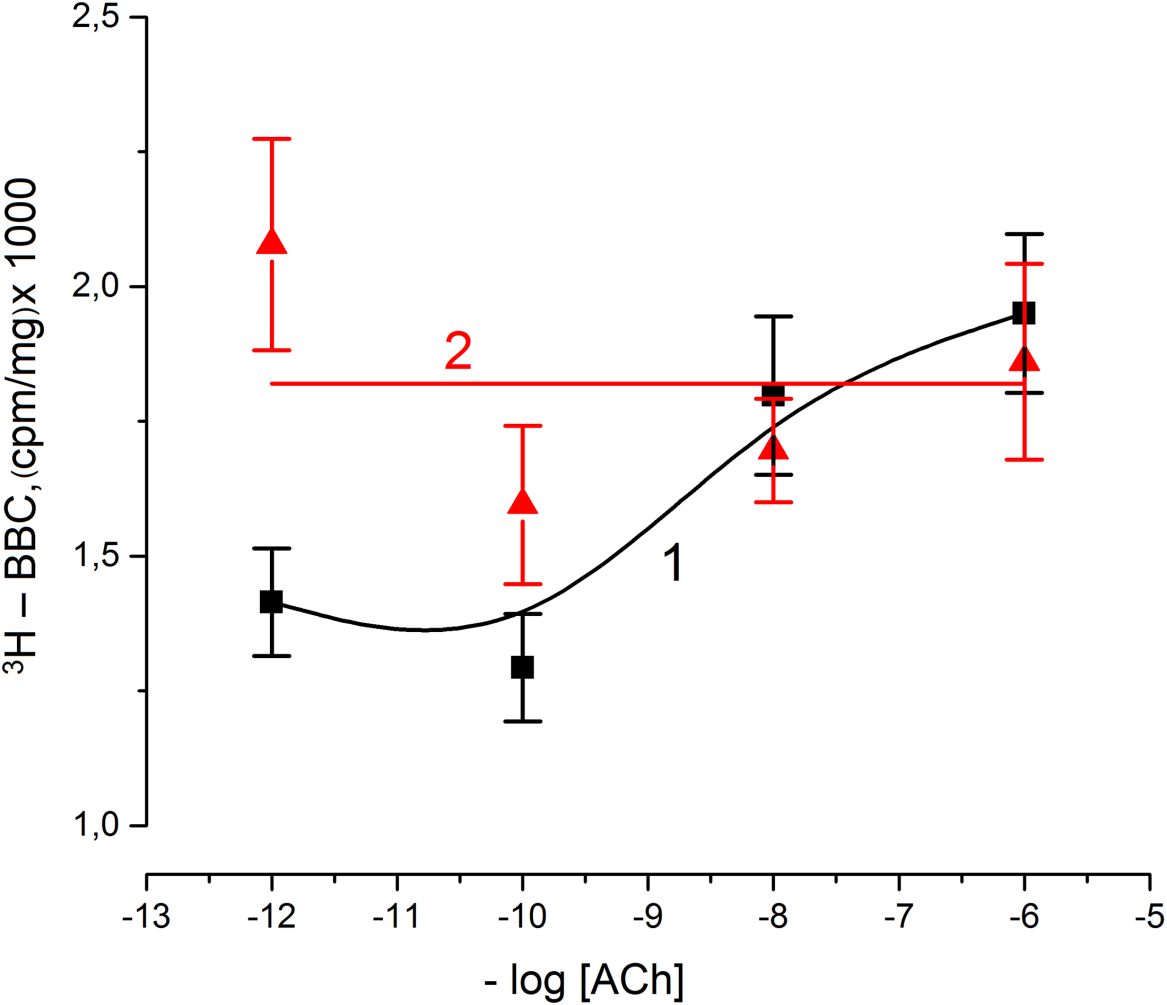
Dependence of specific ^3^H-bungarotoxin binding by Schwann cell non myelin nerve fiber on ACh concentration. Experiments demonstrate specific ^3^H- bungarotoxin binding to Schwann cell non myelin nerve fiber during incubation in following solution: 111 mM NaCl; 2 mM; 1.08 CaCl_2_; 50 mM imidasol- HCl buffer, pH 7,4; 22°C (1, black) and 59 mM NaCl, 52 mM RCl, 1.08 CaCl_2_; 50 mM imidasol- HCl buffer, pH 7,4; 22°C (2, red). The time of nerve fiber ^3^H- bungarotoxin incubation was 10 min; significant difference between points (*p* < 0.05). Here, [ACh]–ACh concentration, BBC—bungarotoxin binding content.

As it was noted earlier, it was established for the giant squid nerve fiber (without myelin) that small ACh concentrations (10^-7^M) induce a long-lasting SC hyperpolarization, due to an increasing permeability of the SC plasma membrane to the K^+^ [4]. According to this, we investigated the SC ^3^H-BT binding dependence on the different extracellular ACh concentrations at high extracellular K^+^ (Fig 2). The maximal ^3^H-BT binding was found at concentrations of ACh in the range 10^−8^–10^−6^ M, but at the axon and SC membrane K^+^-depolarization (nerve fibers were incubated in solution containing 50 mM K^+^), the ^3^H-BT binding was not characterized by sigmoid dependence on the ACh and significant difference between points at 10^−12^ M and 10^−10^ M ACh (p < 0.05)).

In this case, the possible explanations of the observed effects are: (i) the long-lasting axon membrane depolarization stimulated ion channels inactivation and (ii) SC membrane depolarization stimulated AChRs inactivation and blockade of a long-lasting SC hyperpolarization It is known, that increasing of the extracellular K^+^ concentration at axon excitation stimulates the ACh output from SC and affects the receptors [19]. It is possible that during nerve excitation there are increases not only of the single AChRs conductivity, but of their number in SC membrane.

**Fig 3:**
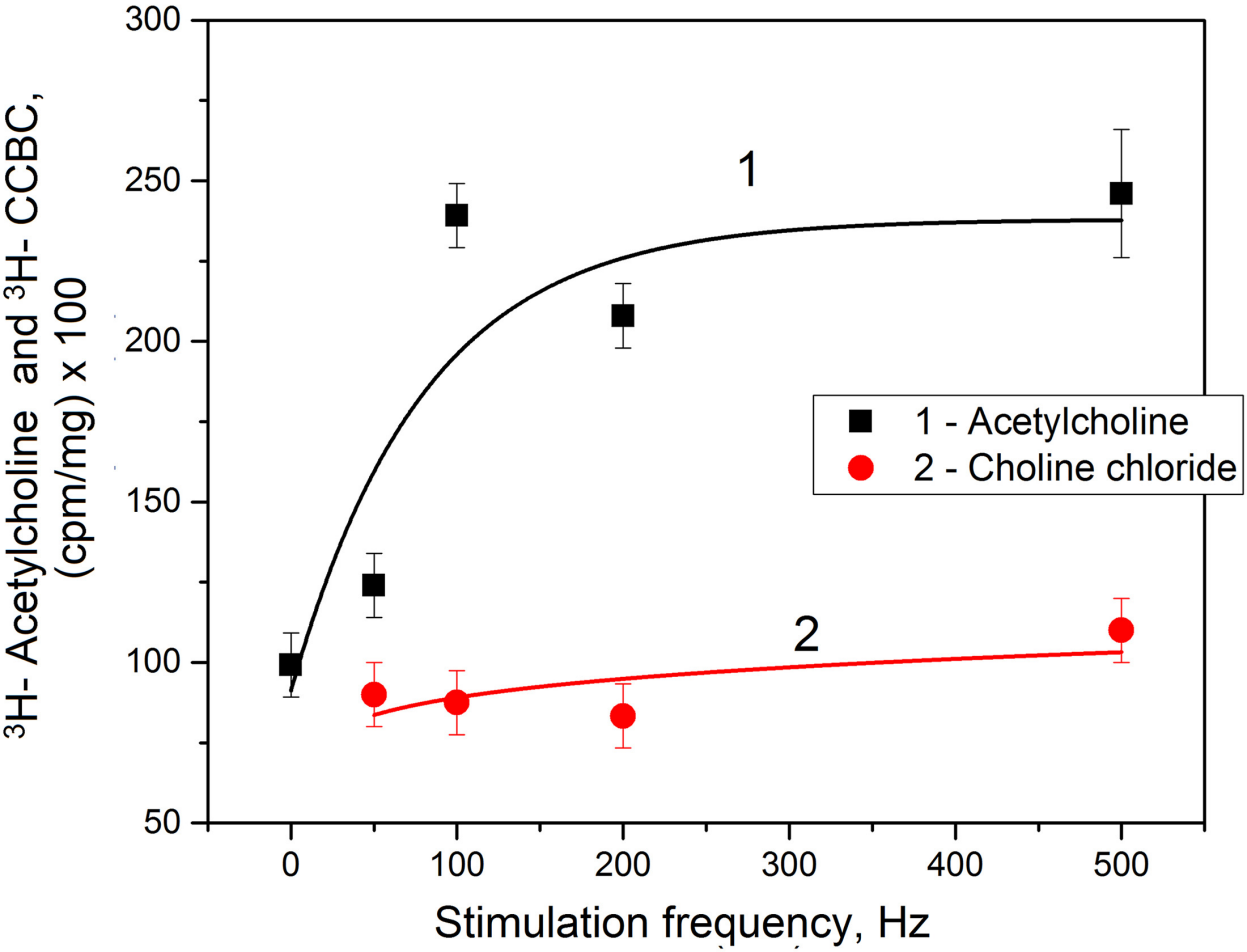
Dependence of specific ^3^H- Acetylcholine (1) and ^3^H-choline chloride (2) binding by Schwann cell myelin nerve fiber on rhythmic excitation frequency. Experiments demonstrate changes in specific ^3^H- Acetylcholine (1) and ^3^H-choline chloride (2) binding by Schwann cell myelin nerve fiber with increasing stimulation frequency. The time of nerve fiber incubation was 60 min, and time of nerve stimulation was 10 min. Here, CCBC—choline chloride binding content. Significant difference between points (*p* < 0.05).

The increase in the number of SC AChRs at axon stimulation could be accompanied by binding of ACh. According to this suggestion, we investigated the binding of labeled ^3^H -ACh during different frequencies of the axon excitation. It was shown that the ^3^H -ACh binding increases with a nerve stimulation frequency and reaches a maximal level at a different number of APs and interval between them (100 Hz frequency) Fig 3). A further decrease of the interval between APs did not affect the ^3^H-ACh binding. It should be noted that a nonspecific binding of the labeled ^3^H- choline chloride has not been detected under the same experimental conditions upon nerve fiber stimulation (Fig 3), which indicates that observed ^3^H-ACh binding is specific. In both cases, the number of APs was equal. Thus, the binding of the labeled ACh and ^3^H-BT with receptors is observed only at the long interval between APs upon axon excitation. During the short interval between APs (> 100 Hz), ^3^H-BT binding decreases as well, and ACh binding is constant at its maximum level. We suggest that such abnormal (non-functional) stimulation (short intervals between APs) was not physiological, thus such conditions are not suitable for the appearance of new “active” receptors on the SC surface. We assume that axon does not conduct AP, as there are no changes in K^+^ concentration in paranodal space, which leads to an increase in the excitation threshold and the transformation of its excitation rhythm, however previously activated ACh receptors retain high affinity and can bind ligand.

Probably, this effect depends on the extracellular concentration of K^+^ in the nerve fibers paranodal space and K^+^- depended Ca^2+^ influx in SC. It is known that at low concentrations of Ca^2+^, the monovalent cations activate the AChRs, but at high extracellular of Ca^2+^ the AChRs transport is due to Ca^2+^ in SC only. It is proposed that in SC about 2% Ca^2+^ influx is connected with AChR and 7% of the total Ca^2+^ through Ca^2+^- channel [19]. In addition, Ca^2+^ modifies the process of the sensitivity of AChR to Ach restore. Therefore, Ca^2+^ influx through AChRs changes during the excitation of the axon, and depolarizes the SC membrane. Thus, the Ca^2+^ flow in the nerve fibers axon increases the number of SC AChRs, but also helps to increase Ca^2+^ influx via AChRs SC channel.

It is possible that during axon excitation the SC AChRs are phosphorylated by cAMP-dependent protein kinase C and protein tyrosine kinase [17,18]. Several studies have indicated that phosphorylation of AChRs correlates with the number of AChR on the cell surface membrane. The forskolin and other agents that increase intracellular cAMP levels increased the number of the fibroblasts AChRs. The activation of the protein kinase C led to an increase in the gamma subunit AChRs lifetime as well as to an increase of the number of AChRs.

### The role of Schwann cell AChR activation in the lipid ordering of the myelin lipids

**Fig 4:**
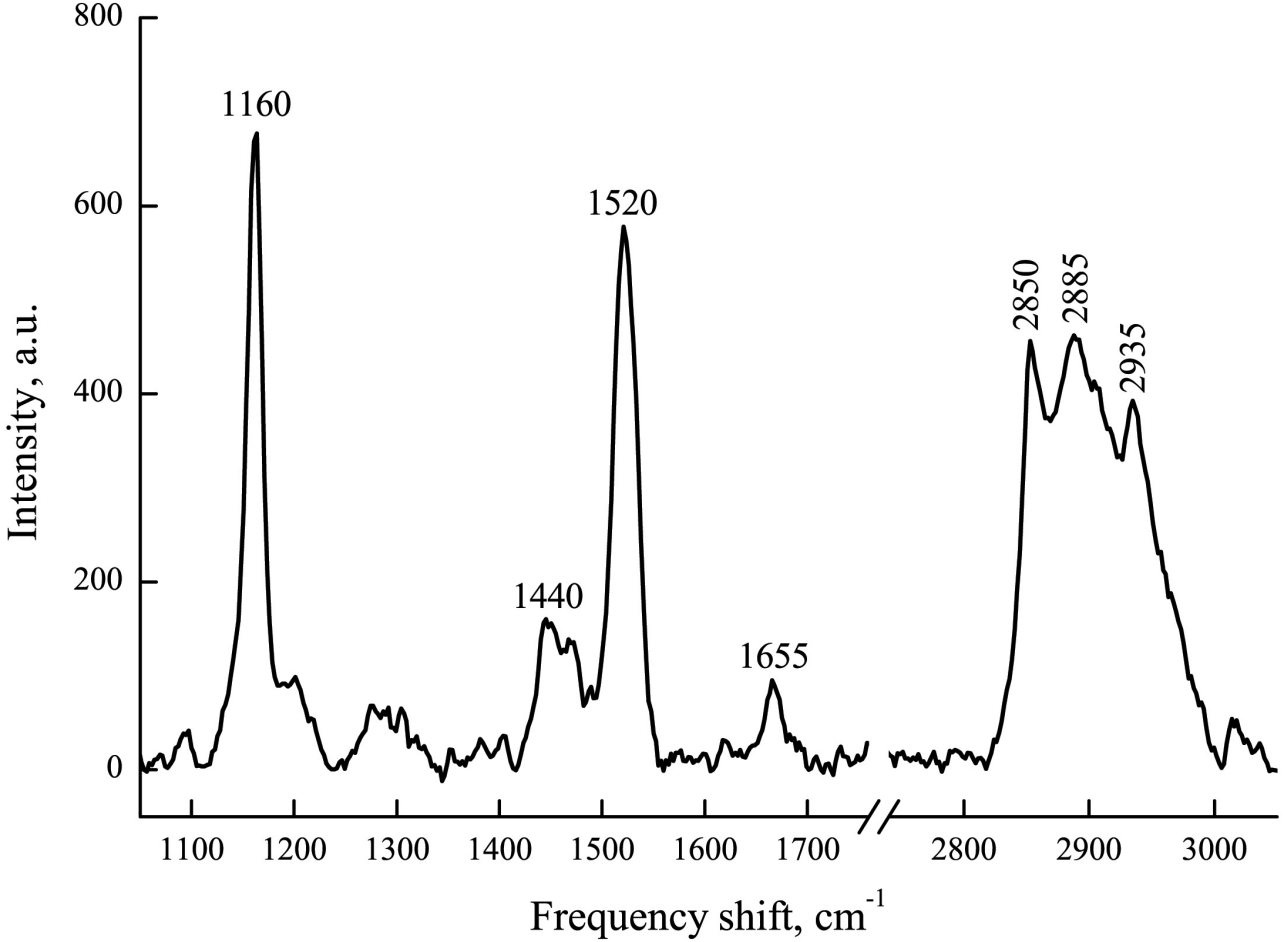
Raman spectra of myelin sheath of sciatic nerve fiber. Raman scattering was excited by 532-nm laser with power 0.8 mW, registration time was 50 s.

In following experiments, the changes of myelin lipids and carotenoids (the minor membrane component) conformation during the long-lasting nerve fibers incubation with ACh were studied. The Raman spectrum of myelin sheath of peripheral nerve fiber could be characterized by presence of a set of intensive bands, related to phospholipid and carotenoid molecules (Fig 4 and Table 1). In this experiment, we will take into consideration only the following RS peaks: 1160, 1520, 2885, and 2935 cm^-1^. As previously shown, carotenoids which are the part of the myelin membrane contribute to the bands at 1160 and 1520 cm^-1^, corresponding to C–C and C = C stretching modes of the polyene chain, respectively. The peaks at 2885 and 2935 cm^-1^ correspond to the methylene C–H asymmetric and methyl C–H symmetric stretching vibrations of the phospholipid acyl chain, respectively [12–14].

**Table 1:** Assignment of main peaks in Raman spectra of myelin sheath of nerve fiber (adopted from [19, 20]).

Frequency shift (cm^-1^)	Characterization of the vibrational mode
1160	C–C stretching vibration of the carotenoid polyene chain
1520	C = C stretching vibration of the carotenoid polyene chain
1445	carbonyl CH_2_ bending vibrational mode of the unsaturated lipid acyl chain
1660	C–C stretching vibration of the unsaturated lipid acyl chain
2850	methylene C–H symmetric stretching vibration of the phospholipid acyl chain
2885	methylene C–H asymmetric stretching mode of the phospholipid acyl chain
2935	terminal methyl group C–H symmetric stretching vibration of the phospholipid acyl chain

**Fig 5:**
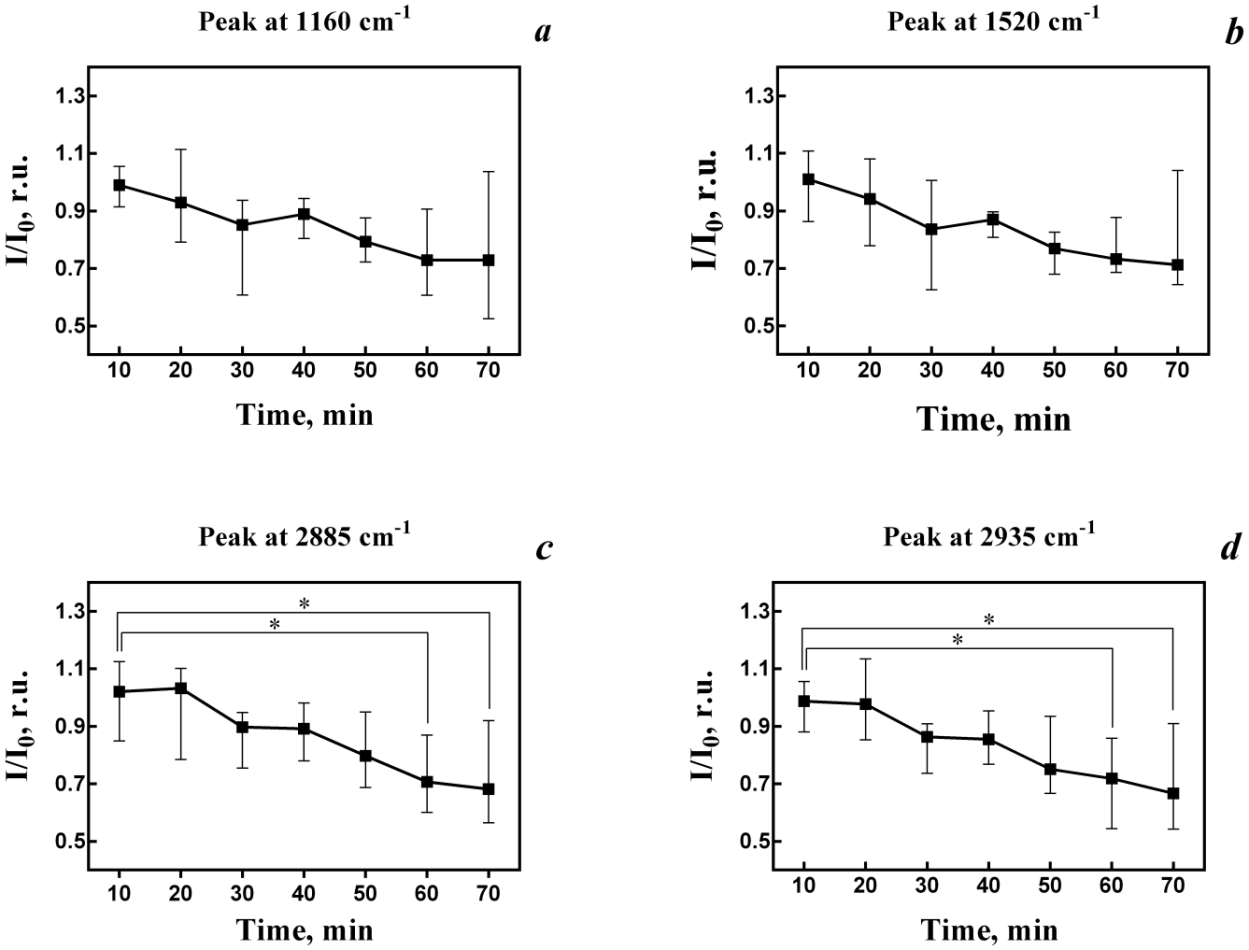
Dynamics of carotenoid (1160 (a) and 1520 (b) cm^-1^) and (2885 (c) and 2935 (d) cm^-1^) peak intensities obtained from Raman spectra of myelin of sciatic nerve fibers. Experiments demonstrate dynamics of carotenoid peak intensities after application of ACh (100 μM). Values of the peak intensities are presented as mean ± SE. Significant difference between time points (*p* < 0.05).

To make sure that there are no dynamic changes in values of the bands intensities, we recorded Raman spectra from myelinated nerve fiber kept for 70 min in Ringer solution (control). Indeed, no changes were observed during the time of the experiment. We examined an effect of ACh (100 μM) on lipid ordering of the myelin membrane (Fig 5). The significant decrease of the lipid RS peak intensities was observed mostly after 50 min ACh action. Observed changes of intensities of the RS peaks at 2885 and 2935 cm^-1^ correspond to a reduction in lipid fat acids bond vibrations after application of ACh (Fig 5c and 5d). In other terms, application of ACh led to an increase in degree of lipid ordering of the myelin membrane. So, we found that during prolonged ACh incubation of nerve fibers the changes of intensities of the peaks at 2935 cm^-1^ and 2885 cm^-1^ occur at long time (60 and 70 min) due to changes in fatty tails of phospholipids ordering.

It is known that the carotenoid molecules significantly influence on the phase transition of the lipid myelin membrane, because it may increase the local order of fatty acids "tails" [19, 20]. We assume that areas of the lipid bilayer containing carotenoids and form “rafts” may bind two separate bilayers. In our case, the activation of SC AChRs only affects the ordering of the membrane phospholipids, without changing molecular conformation of carotenoids in the "rafts". It is known that the inactivation of the SC enzyme—phospholipase A_2_ increase the speed of lateral diffusion of lipids [21, 22]. Perhaps SC AChRs activation, followed by Ca^2+^ influx and stimulation of the Ca^2+^- dependent phospholipase activity, can change the viscosity of nerve fibers myelin lipids.

## Conclusions

We can conclude that a high electrical resistance of myelin, which is important for conduction of a series of action potentials, not only depends on the lipid composition, but can be also regulated by activation of AChRs upon the interaction of axon and SC.
